# Remarkable Response to Immune Checkpoint Inhibition After Early Aggressive Recurrence Following Robot‐Assisted Radical Cystectomy for Bladder Squamous Cell Carcinoma: A Case Report

**DOI:** 10.1002/iju5.70252

**Published:** 2026-08-18

**Authors:** Kazuma Soya, Takeru Fujimoto, Yu Osaki, Koji Matsumoto, Yusuke Takei, Yoshio Sugino, Tsutomu Maruta, Hiroshi Iwamura

**Affiliations:** ^1^ Department of Urology National Hospital Organization Himeji Medical Center Himeji Hyogo Japan; ^2^ Department of Medical Oncology Hyogo Cancer Center Akashi Hyogo Japan; ^3^ Department of Diagnostic Pathology National Hospital Organization Himeji Medical Center Himeji Hyogo Japan; ^4^ Department of Radiology National Hospital Organization Himeji Medical Center Himeji Hyogo Japan

**Keywords:** bladder cancer, pembrolizumab, squamous cell carcinoma

## Abstract

**Introduction:**

Squamous cell carcinoma is a rare bladder cancer subtype with poor prognosis in advanced stages. Currently, there is no established systemic therapy, including immune checkpoint inhibitors, for this subtype.

**Case Presentation:**

A 49‐year‐old man with pure bladder squamous cell carcinoma arising from a diverticulum with pelvic lymph node involvement underwent neoadjuvant gemcitabine plus cisplatin followed by robot‐assisted radical cystectomy. Six months later, pulmonary metastases developed, and platinum rechallenge led to rapid progression with pleural dissemination. Pembrolizumab produced a complete response despite programmed cell death ligand‐1 negativity, microsatellite stability, and low tumor mutational burden. Isolated hilar lymph node progression during treatment was managed with radiotherapy, achieving temporary local control. The patient survived 41 months from diagnosis and 30 months after metastasis.

**Conclusion:**

Despite early and aggressive metastatic recurrence after robot‐assisted radical cystectomy, the patient showed a remarkable and durable response to pembrolizumab.

## Keynote Message

We report a case of bladder squamous cell carcinoma that recurred early and aggressively after robot‐assisted radical cystectomy yet responded remarkably and durably to pembrolizumab despite negative conventional biomarkers. This case highlights the potential role of immune checkpoint inhibitors and the need for awareness of early recurrence in high‐risk disease.

**Abbreviations**
GC: gemcitabine plus cisplatin
ICIs: immune checkpoint inhibitors
MSI: microsatellite instability
PD‐L1: programmed cell death ligand‐1
RT: radiotherapy
SCC: squamous cell carcinoma
TMB: tumor mutation burden

## 1 Introduction

Squamous cell carcinoma (SCC) of the bladder is a rare histological subtype, accounting for 1%–5% of bladder cancers [1]. Surgical resection is the mainstay for localized disease, as bladder SCC is generally resistant to radiotherapy (RT) and conventional chemotherapy. Owing to its rarity, no standard systemic therapy has been established, and the prognosis for advanced disease remains poor [2]. Evidence supporting the efficacy of immune checkpoint inhibitors (ICIs) in bladder SCC is limited.

We report a case of bladder SCC that recurred early and aggressively after robot‐assisted radical cystectomy (RARC) but showed a remarkable and durable response to pembrolizumab.

## 2 Case Report

**FIGURE 1:**
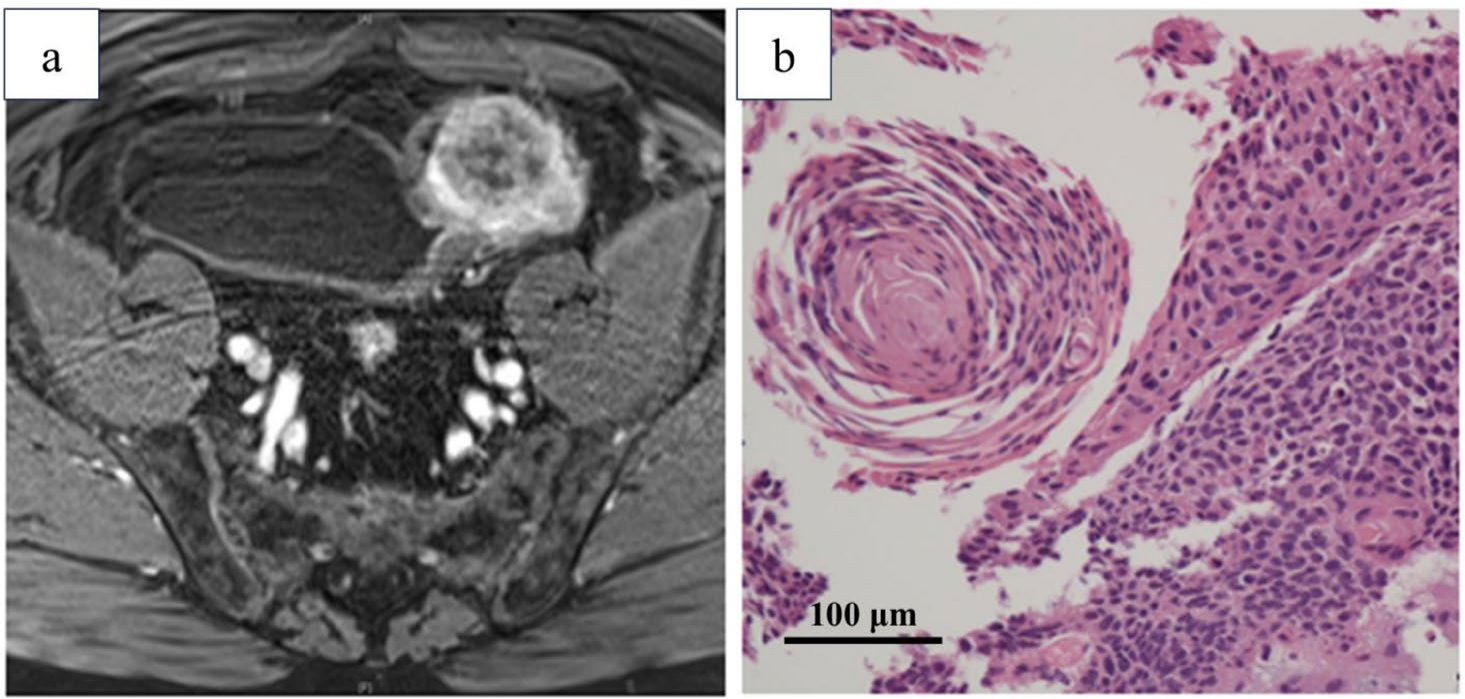
Imaging and pathological findings before radical cystectomy (a) Magnetic resonance imaging showing a mass in the left lateral wall diverticulum of the bladder, which had infiltrated beyond the bladder wall. (b) Pathology specimen from transurethral resection of the bladder tumor. Hematoxylin and eosin staining reveals spindle‐shaped atypical cells with keratinization proliferating in layers and infiltrating the tissue.

A 49‐year‐old man presented with dysuria and gross hematuria. Imaging revealed a tumor arising from a bladder diverticulum with extravesical invasion and bilateral obturator lymphadenopathy (cT3bN2M0; Figure 1a). Transurethral resection confirmed the histology of pure SCC with muscle invasion (Figure 1b).

The patient received neoadjuvant gemcitabine plus cisplatin (GC), achieving stable disease in the primary tumor and partial response in lymph nodes. He subsequently underwent RARC with ileal neobladder reconstruction. Pathology showed well‐differentiated pure SCC (ypT3a, ypN0, G3, INFb, ly1, v0, u‐lt0, u‐rt0, ur0, and RM0).

**FIGURE 2:**
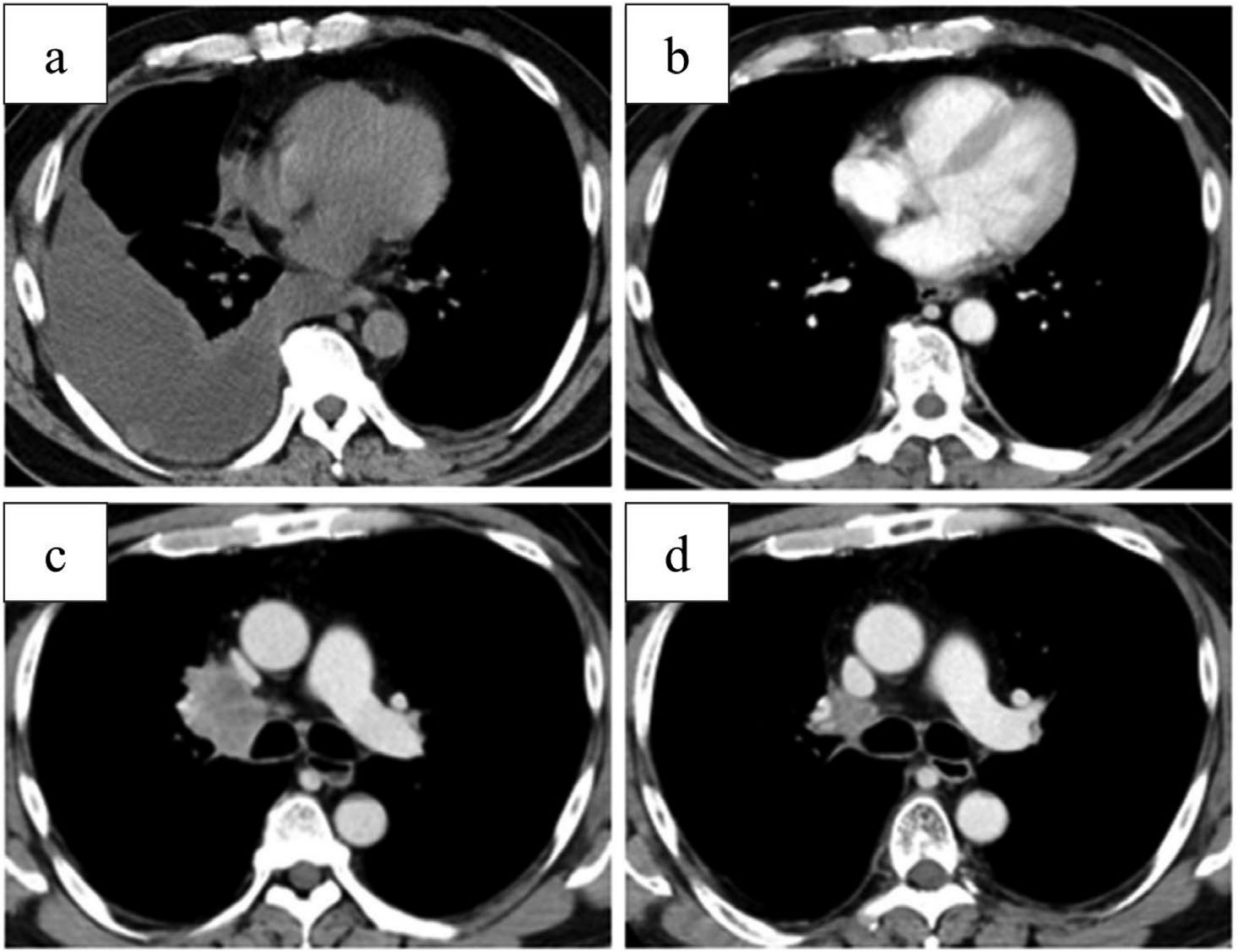
Postoperative imaging findings following gemcitabine and cisplatin chemotherapy for recurrence after radical cystectomy, which proved ineffective (a) Computed tomography (CT) just before initiating pembrolizumab therapy, revealing massive pleural effusion accumulation, suggesting progression to cancerous pleurisy caused by pleural dissemination. (b) CT at 6 months after the start of pembrolizumab: Complete response was achieved. (c, d) CT at 15 months after the beginning of pembrolizumab: an enlarged lymph node appeared in the right hilar region. It was determined to be oligoprogressive, and radiation therapy was administered, resulting in tumor shrinkage.

Six months later, multiple pulmonary metastases were detected and confirmed histologically. Because a certain therapeutic effect was observed with preoperative chemotherapy, GC was reintroduced but resulted in progressive disease, accompanied by massive pleural effusion and dyspnea (Figure 2a). Pembrolizumab was initiated as second‐line therapy. Comprehensive genomic profiling revealed microsatellite stability and a tumor mutation burden (TMB) of 6 muts/Mb, with no other treatment‐eligible gene mutations identified.

After 1.5 months, pleural effusion decreased and symptoms improved, although pleural nodules initially enlarged. Considering possible pseudoprogression, treatment was continued, leading to further tumor regression and eventual complete response at 6 months (Figure 2b).

Pembrolizumab was maintained; however, 12 months later, isolated progression of a right hilar lymph node was observed, consistent with oligoprogression, which was associated with cough (Figure 2c). Surgical resection was not feasible due to adhesions, and RT (60 Gy in 30 fractions) was administered, resulting in tumor shrinkage and symptom relief (Figure 2d).

Three months after RT, grade 2 radiation pneumonitis occurred, and pembrolizumab was discontinued. Six months after RT, nodal regrowth with bronchial obstruction, pleural effusion, and pneumonia developed. The patient declined further systemic therapy and opted for best supportive care; he died 41 months after diagnosis (30 months after metastasis).

Retrospective analysis of the lung metastasis that responded to pembrolizumab showed programmed cell death ligand‐1 (PD‐L1) tumor proportion score < 1%. Serum SCC antigen was normal (1.4 ng/mL; reference < 2.0) immediately after RARC, rose slightly (4.6 ng/mL) at pulmonary metastasis, and remained stable thereafter, thus not reflecting the clinical course.

## 3 Discussion

This case illustrates three noteworthy features of advanced bladder SCC: the early aggressive metastasis after RARC, the marked response to ICIs despite negative conventional biomarkers, and the role of radiotherapy in subsequent oligoprogression.

Bladder SCC is a rare subtype of bladder cancer that typically arises in the setting of chronic irritation and inflammation [2]. Radical surgery is the mainstay for localized disease [3]; however, the efficacy of pre‐ and postoperative adjuvant chemotherapy remains unclear. This histology is generally resistant to radiotherapy and platinum‐based chemotherapy [1], and no evidence‐based systemic therapy has been established. Consequently, the prognosis remains poor, with most patients dying within 3 years and a 5‐year survival rate of 33%–48% [2].

In this case, pulmonary metastases developed within 6 months of cystectomy and progressed rapidly to pleural dissemination. Given the high tumor stage (ypT3a) and pure squamous histology, intrinsic tumor aggressiveness is one plausible explanation for early systemic progression. The surgical approach may also have contributed. Although the RAZOR trial found RARC non‐inferior to open surgery for progression‐free survival without increasing early recurrence [4], atypical recurrences after RARC—typically peritoneal, extra‐pelvic nodal, or port‐site—have been postulated to relate to pneumoperitoneum‐associated tumor spread, with high‐stage disease as a risk factor [5, 6]. Our patient had such features, so this cannot be excluded. However, larger analyses attribute such recurrences to tumor biology rather than the pneumoperitoneum itself [6], and the lung‐to‐pleura pattern here differs from spillage‐related recurrence, instead supporting hematogenous dissemination. This case underscores that high‐stage, aggressive bladder SCC remains at risk of early recurrence even after R0 resection, warranting vigilant surveillance.

Following the failure of platinum‐based chemotherapy, pembrolizumab was initiated as second‐line therapy. Evidence regarding the efficacy of ICIs in bladder SCC is extremely limited, with only two previously reported cases: one treated with pembrolizumab for pelvic recurrence after cystectomy [7] and another with tislelizumab for locally advanced disease [8]. A summary of the cases is presented in Table 1. Several biomarkers have been proposed to predict ICI response, including PD‐L1 expression, microsatellite status, and TMB [9]. PD‐L1 expression has been associated with ICI efficacy in various SCCs [10, 11, 12, 13] and urothelial carcinoma [14], and is reportedly more frequent in bladder SCC than in urothelial carcinoma [15]. Consistent with this, the two previously reported ICI‐responsive cases showed PD‐L1 positivity. In contrast, our case demonstrated a marked response despite PD‐L1 negativity in metastatic lesions. Furthermore, microsatellite instability (MSI) was absent, and the TMB was low, neither suggesting high sensitivity to ICIs. ICI benefit in other‐organ SCCs is not confined to PD‐L1–positive disease. In squamous non–small cell lung cancer, second‐line nivolumab improved overall survival irrespective of PD‐L1 expression, including at PD‐L1 < 1% [10]. A similar, though nonsignificant, benefit was suggested in the PD‐L1 < 1% subgroups of esophageal [11], head and neck [12], and cervical [13] SCC (Table 2). These benefits were observed without selection for MSI or TMB, consistent with the marked response in our PD‐L1–negative, microsatellite‐stable, low‐TMB tumor. These findings highlight the limitations of current biomarkers in predicting ICI response and underscore the need for further clinical evidence and biomarker development in bladder SCC.

**TABLE 1:** A summary of cases of urinary bladder squamous cell carcinoma responding to immune checkpoint inhibitors.

Author, year	Kao, 2019	Dong, 2025	Present case
Sex	Male	Male	Male
Age	63	45	49
Immune checkpoint inhibitor (ICI)	Pembrolizumab	Tislelizumab	Pembrolizumab
Prior systemic therapy before ICI	None	Gemcitabine‐Cisplatin	Gemcitabine‐Cisplatin
Metastatic sites at ICI initation	Pelvic lymph node	None	Lung, Pleura
Best responce during ICI treatment	Complete response	Complete response	Complete response
Outcome	NED at 26 months	NED at 28 months	DOD at 41 months
PD‐L1 status	Positive (TPS = 60%–70%)	Positive (CPS > 1)	Negative (TPS < 1%)
Microsatellite (MS) status	MS‐Stable	N/A	MS‐Stable
Tumor mutationnal burden	N/A	N/A	6 Muts/Mb

Abbreviations: CPS, combined positive score; DOD, dead of disease; ICI, immune checkpoint inhibitor; Mb, Megabase; MS, microsatellite; Muts, mutations; N/A, not applicable; NED, no evidence of disease; PD‐L1, programmed cell Death‐ligand 1; TPS, tumor proportion score.

**TABLE 2:** Immune checkpoint inhibitor therapy in squamous cell carcinomas of other organs: Overall survival and outcomes in the PD‐L1 < 1% subgroup.

SCC site (Trial) [ref]	ICI (line)	Median OS, ICI vs. control (overall HR, 95% CI)	HR in PD‐L1 < 1% (95% CI)
Lung (CheckMate 017) [10]	Nivolumab (2L)	9.2 vs. 6.0 months (HR 0.59, 0.44–0.79)	0.58 (0.37–0.92)
Esophagus (ATTRACTION‐3) [11]	Nivolumab (2L)	10.9 vs. 8.4 months (HR 0.77, 0.62–0.96)	0.84 (0.62–1.14)
Head and neck (CheckMate 141) [12]	Nivolumab (2L)	7.5 vs. 5.1 months (HR 0.70, 0.51–0.96)	0.89 (0.54–1.45)
Cervix (EMPOWER‐Cervical 1) [13]	Cemiplimab (2L)	11.1 vs. 8.8 months (HR 0.73, 0.58–0.91)^a^	0.98 (0.59–1.62)^b^

Abbreviations: 2L, second‐line; CI, confidence interval; HR, hazard ratio; ICI, immune checkpoint inhibitor; OS, overall survival; PD‐L1, programmed death‐ligand 1; SCC, squamous cell carcinoma.

^a^ Data in the squamous cell carcinoma subgroup.

^b^ The PD‐L1 < 1% subgroup was not stratified by histology (squamous or adeno/adenosquamous).

During pembrolizumab therapy, isolated progression of a hilar lymph node was consistent with oligoprogression, and local treatment was considered appropriate in the absence of alternative systemic options. Treatment was planned according to the principles of primary tumor management, in which surgery was the first choice. However, the lesion was unresectable due to extensive adhesions; therefore, RT was administered. This provided symptom relief and temporary disease control, with a progression‐free interval of approximately 6 months. This interval was shorter than the median progression‐free survival of 7–12 months reported after metastasis‐directed radiotherapy for oligoprogressive disease in other malignancies [16, 17], possibly reflecting the limited radiosensitivity of bladder SCC. Given this single case, no conclusion can be drawn regarding metastasis‐directed therapy, and further cases are needed to clarify its role.

## 4 Conclusion

We report a case of advanced pure bladder SCC that recurred early and aggressively after RARC, yet responded markedly and durably to pembrolizumab despite negative conventional biomarkers. This highlights the potential role of ICIs in this rare malignancy lacking established systemic therapy and the need for awareness of early recurrence.

## Consent

Informed consent was obtained from the patient for publication of this case report.

## Conflicts of Interest

The authors declare no conflicts of interest.

## Data Availability

Data sharing not applicable to this article as no datasets were generated or analysed during the current study.
